# Successful reintegration of a very elderly patient with rapidly progressive glomerulonephritis due to microscopic polyangiitis after multidisciplinary treatment: a case report

**DOI:** 10.3389/fneph.2026.1803876

**Published:** 2026-04-28

**Authors:** Tatsuo Tsukamoto, Yui Fukuda, Kaoru Ohue, Atsuto Niwa, Sanae Ogura, Takuya Ishimura, Takaya Handa, Tomomi Endo, Takeshi Matsubara

**Affiliations:** 1 Department of Nephrology and Dialysis, Medical Research Institute Kitano Hospital, PIIF Tazuke-Kofukai, Osaka, Japan; 2 Center for Preventive Medicine, Medical Research Institute Kitano Hospital, PIIF Tazuke-Kofukai, Osaka, Japan

**Keywords:** elderly population, microscopic polyangiitis, multidisciplinary approach, rapidly progressive glomerulonephritis, reintegration

## Abstract

ANCA (anti-neutrophil cytoplasmic antibody)-associated vasculitis (AAV) is known to cause rapidly progressive renal dysfunction, as well as high mortality, especially in older patients. In Japan, a high prevalence of microscopic polyangiitis (MPA) with myeloperoxidase (MPO)-ANCA among older patients has been reported; however, standard immunosuppressive therapy does not always lead to improved outcomes in this population. Here we report the case of a 93-year-old woman who developed rapidly progressive glomerulonephritis (RPGN) due to MPA and successfully returned home after multidisciplinary treatment. She was transferred from another hospital because of antibiotic-refractory fever. Serological testing revealed MPO-ANCA positivity and a renal biopsy demonstrated crescentic glomerulonephritis. She was diagnosed with RPGN due to MPA and was initiated on standard glucocorticoid therapy, despite her advanced age. Because of severe disease activity complicated by diffuse alveolar hemorrhage, noninvasive positive pressure ventilation, rituximab, avacopan, and plasma exchange combined with hemodialysis were introduced. This multidisciplinary approach successfully induced remission of her AAV with partial recovery of renal function. After rehabilitation for mobilization, the patient was discharged home. She currently attends outpatient follow-up independently without assistance. Although very elderly patients are often excluded from clinical trials, resulting in limited evidence regarding the efficacy and safety of therapeutic agents, appropriately dose-adjusted multidisciplinary treatment based on overall clinical conditions may improve outcomes, even in this population.

## Introduction

Rapidly progressive glomerulonephritis (RPGN) caused by ANCA (anti-neutrophil cytoplasmic antibody)-associated vasculitis (AAV) leads not only to severe renal dysfunction requiring kidney replacement therapy but also to life-threatening systemic organ damage due to widespread vasculitis (1). Therefore, prompt initiation of intensive immunosuppressive therapy and careful disease monitoring are essential (2, 3). Several therapeutic agents developed recently have contributed to improving mortality; however, they have not shown satisfactory improvement of renal outcome to the same extent (1). A characteristic feature of AAV in Japan is its predominance among older patients compared with patients in Western countries (4–6). In particular, a high prevalence of microscopic polyangiitis (MPA) with myeloperoxidase (MPO)-ANCA among older patients has been reported to be significant in Japan (5–7). Older patients are frequently excluded from clinical trials of newly approved therapies, resulting in a paucity of evidence regarding their efficacy and safety in this population (8). Therefore, in the treatment of AAV in the very elderly, who have reduced drug metabolism and increased susceptibility to infection with multiple comorbidities, it is necessary to combine various treatment options for rapid remission induction and maintenance of AAV, in addition to dose adjustments of therapeutic agents for better outcomes.

Here, we report the case of a very elderly patient who presented with RPGN and diffuse alveolar hemorrhage (DAH) due to MPA. She was successfully treated with multidisciplinary therapy including immunosuppressive therapy, plasma exchange (PLEX) with hemodialysis, and noninvasive positive pressure ventilation (NPPV), which ultimately enabled her to return home with minimal care support.

## Case description and diagnostic assessment

**Figure 1:**
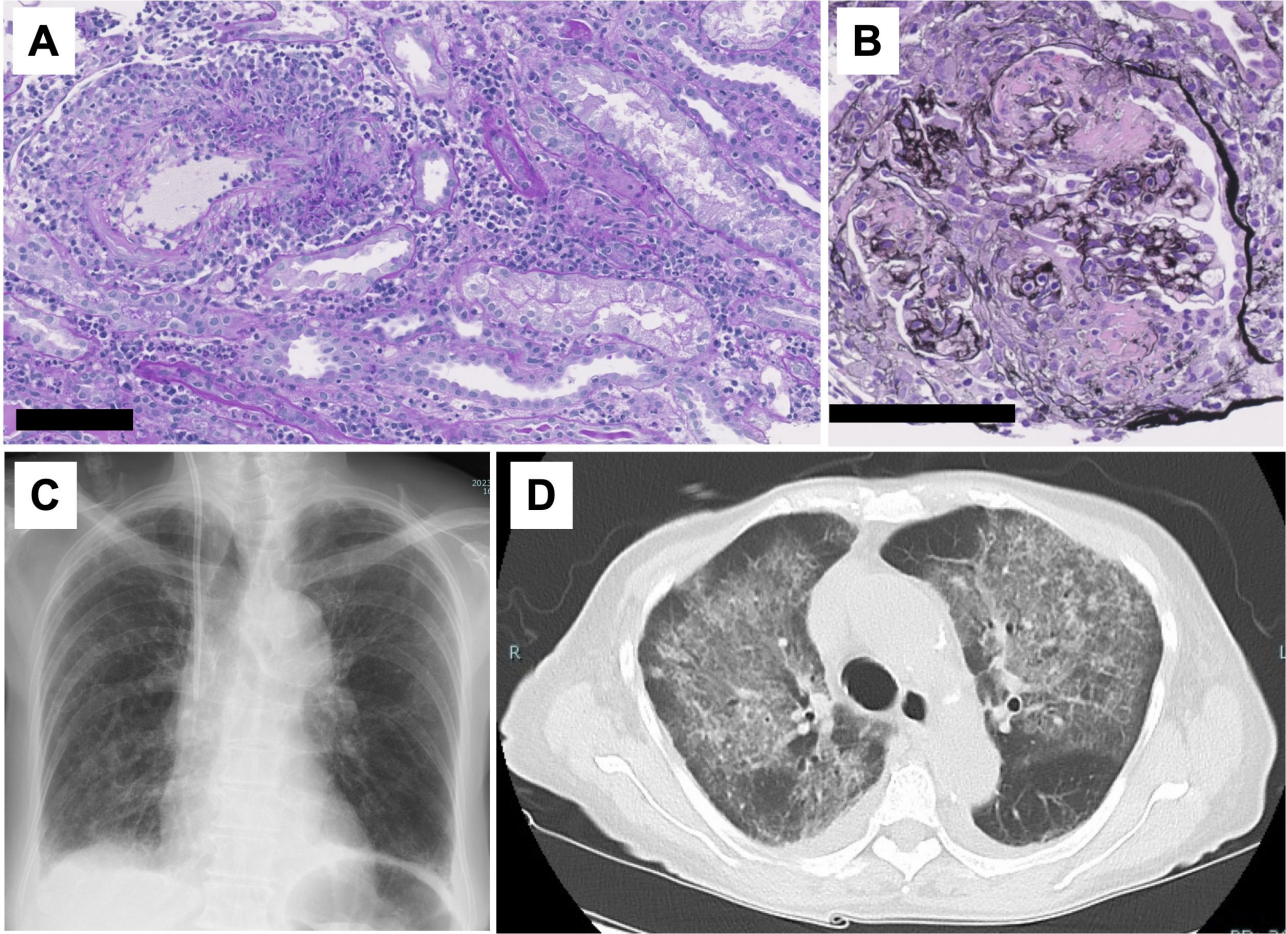
Renal biopsy reveals crescentic glomerulonephritis. Three glomeruli show globally sclerosis and five have cellular or fibrocellular crescent out of a total of 14 glomeruli. Of the 14 glomeruli collected, 3 showed global sclerosis (21.4%), 5 possessed cellular or fibro-cellular crescents (35.7%), and 1 had fibrous crescents. **(A)** Necrotic lesions in the interstitial arterioles and peritubular capillaritis were also observed (periodic acid Schiff; PAS staining; bar 100 μm). **(B)** Most affected glomerulus possessed fibro-cellular crescents (Periodic Acid Methenamine Silver; PAM staining; bar 100 μm). **(C)** Chest X-ray on hemoptysis. **(D)** Segmental increase of interstitial shadow was observed in both lungs, consistent with alveolar hemorrhage.

**Figure 2:**
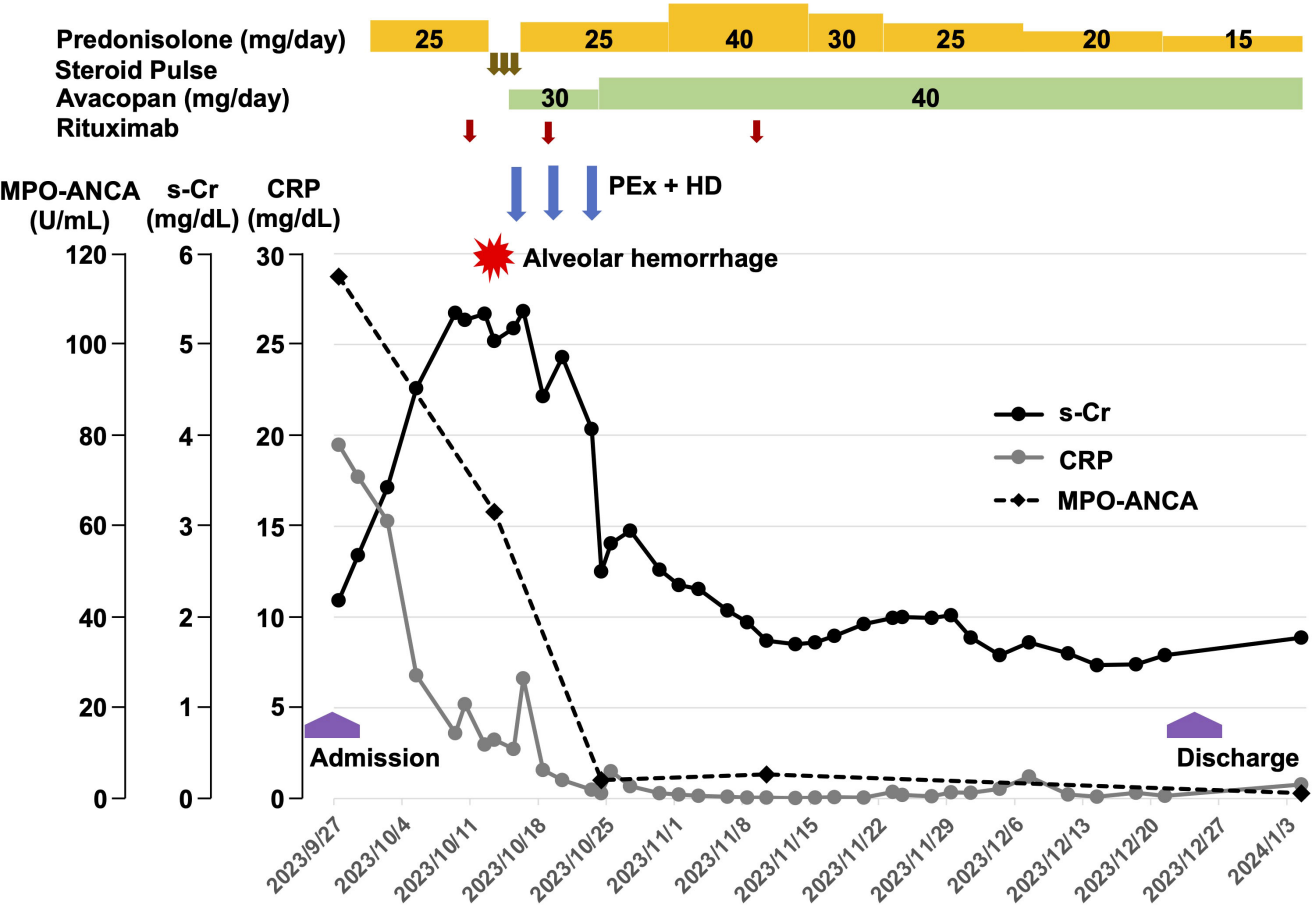
Clinical course of the case.

A 93-year-old woman was transferred from another hospital because of fever of unknown origin, accompanied by rapidly progressive renal dysfunction. She had also been treated for hypertension and non-sustained atrial fibrillation, and had undergone bilateral hip replacement surgery. Prior to disease onset, she had lived independently with her family. On admission, her height was 153.9 cm, body weight 58.4 kg, and body temperature 37.9 °C. Heart and lung sounds were normal and her abdomen was unremarkable. Although mild bilateral lower leg edema was present, neurological abnormalities were not found. Despite prior antibiotic therapy, her fever persisted. Laboratory tests showed microscopic hematuria, persistent proteinuria, marked systemic inflammation, anemia and renal failure (**Table 1**). Her kidney function before the onset was unknown, but her serum creatinine (s-Cr) was 0.82 mg/dL (eGFR 50 mL/min/1.73m^2^) 10 years ago. There was no significant disease before this admission. Serological testing revealed elevated MPO-ANCA levels. A renal biopsy performed on hospital day 5 demonstrated pauci-immune crescentic glomerulonephritis; Of the 14 glomeruli collected, 3 showed global sclerosis (21.4%), 5 possessed cellular or fibro-cellular crescents (35.7%), and 1 had fibrous crescents. Necrotic lesions in the interstitial arterioles and peritubular capillaritis were also observed, leading to the diagnosis of RPGN due to MPA (**Figures 1A, B**). Oral prednisolone at 25 mg once a day (0.4mg/kgBW) was first initiated on hospital day 6. We subsequently determined that the disease activity of her MPA was quite high (Birmingham Vasculitis Activity Score; BVAS 20 points), however, the chronic damage on biopsy specimen was limited (9–11). Taken together, she was a candidate for the standard therapy for MPA based on those clinical factors other than her age. Regarding the potential organ reversibility, functional reserve, and risk of treatment-related adverse events, we explained the disease condition as well as the therapeutic goal to our patient and her family in accordance with the process of shared-decision making. Because renal biopsy showed predominantly active crescentic lesions with limited chronic damage, partial renal recovery was considered possible. In addition, the patient had been functionally independent before admission and had no dementia, although treatment-related risks such as infection and delirium were carefully considered. After discussion with the patient and her family, including the goal of possible return to home life, we chose an individualized remission-induction strategy despite her advanced age. We had to control her urinary tract infection before intensifying an immunosuppression therapy with higher dose of glucocorticoid and rituximab. We decided to use rituximab before dose escalation of her glucocorticoids. We prescribed rituximab 580 mg (375 mg/m²) on hospital day 16 after confirming that the infection had subsided (**Figure 2**).

**Table 1:** Laboratory data on admission.

Urinalysis			Blood chemistry		
Protein	2+		AST	30	U/L
Occult blood	3+		ALT	10	U/L
Urobilinogen	±		LDH	169	U/L
Gradient	1.015		ALP	87	U/L
RBC	>100	/HPF	g-GTP	41	U/L
WBC	50-99	/HPF	T-Bil	0.6	mg/dL
NAG	60.5	IU/L	CK	17	U/L
uPCR	1425.6	mg/gCr	Glucose	105	mg/dL
CBC			Total Protein	5.7	g/dL
RBC	341	x10^4^/mL	Albumin	1.8	g/dL
Hb	9.5	g/dL	T-Chol	132	mg/dL
Ht	29.2	%	LDL-Chol	58	mg/dL
WBC	12,800	/mL	BUN	27.6	mg/dL
PLT	28.4	x10^4^/mL	UA	8.1	mg/dL
Immunology			Cr	2.18	mg/dL
IgG	1297	mg/dL	Na	133	mEq/L
IgA	484	mg/dL	K	3.7	mEq/L
IgM	62	mg/dL	Cl	97	mEq/L
C3	97	mg/dL	Ca	7.8	mg/dL
C4	17	mg/dL	P	3.5	mg/dL
MPO-ANCA	115	U/mL	CRP	19.49	mg/dL
PR3-ANCA	<1.0	U/mL			
anti-GBM Ab	<2.0	U/mL			
ANA	x40				

PCR, protein creatinine ratio.

On hospital day 18, she developed productive cough and hemoptysis. Chest computed tomography showed increased diffuse interstitial infiltrates in her bilateral lungs (**Figures 1C, D**). Although there was no significant progression of anemia (hemoglobin 8.7g/dL), we diagnosed probable DAH based on CT images and hemoptysis. Bronchoscopy was not performed in consideration of the patient’s general condition. As she also developed hypoxia, a noninvasive positive pressure ventilation (NPPV) was initiated. High-dose intravenous methylprednisolone pulse therapy (1,000 mg daily for 3 days) with avacopan (30 mg/day; 20 mg after breakfast and 10 mg after dinner) was started. PLEX, directly combined in series with hemodialysis (HD) was performed three times to control disease activity as a rapid remission induction strategy (12). The substitution solution of PLEX was one plasma volume of fresh frozen plasma (FFP). HD was applied to reduce the burden of large amounts of sodium citrate contained in FFP and to regulate her body fluid volume. Nafamostat mesilate (30 mg/hour) was used for anticoagulation. Since sufficient urine volume was obtained, we did not perform additional HD after that. On hospital day 27, fever recurred and urine culture identified the presence of *Pseudomonas aeruginosa*. This infection was successfully treated with antibiotics and prednisolone was increased to 40 mg once a day on hospital day 35. Her glucocorticoid administration was subsequently tapered in accordance with the vasculitis activity (**Figure 2**).

During NPPV therapy, she developed mild delirium associated with insomnia and aerophagia-induced abdominal pain. Minimal sedative drugs were used to avoid respiratory suppression. The PEXIVAS trial also reported earlier remission induction with the addition of PLEX, we conducted PLEX with HD for quick reduction of MPO-ANCA levels (12). During NPPV therapy, she developed mild delirium associated with insomnia and aerophagia-induced abdominal pain. Minimal sedative drugs were used to avoid respiratory suppression. PLEX with HD was added as part of multidisciplinary treatment for rapid reduction of MPO-ANCA levels, after which her general condition, including hypoxemia, improved (**Figure 2**). Glucocorticoid tapering was performed according to the rapid tapering method described in the PEXIVAS (plasma exchange and glucocorticoid dosing in the treatment of anti-neutrophil cytoplasm antibody-associated vasculitis) protocol (13). As avacopan safety in the very elderly was unknown, avacopan was started at 30 mg/day (14). After confirmation of her liver stability, this dose was subsequently increased to 40 mg/day (15). We did not increase avacopan from 40 mg daily to 60 mg, a fixed dose in ADVOCATE study, after achieving remission of MPA (16). A standard dose of rituximab was used three times without any adverse effects on hospital day16, 23 and 43 (**Figure 2**) (17). The standard administration method for rituximab has been reported to be at a dose of 375 mg/m^2^ once weekly for 4 weeks (18). In our case, the second dose of rituximab was administrated one week after the first dose. The third dose was skipped due to urinary tract infection as described. We used rituximab on hospital day 43 (the fourth week) after her urinary tract infection was subsided. At this time, the MPO-ANCA became negative. Since a single dose of rituximab have induced remission of MPA, we discontinued rituximab treatment after a total of three doses (19). As her oxygen demands decreased, earlier rehabilitation for mobilization was initiated. Although she had severe renal dysfunction, no particular dietary restrictions were imposed during her hospital days. Finally, she was discharged home on hospital day 87.

Support services for outpatient visits after her discharge gradually became unnecessary. Six months after discharge, she was able to visit our outpatient clinic independently. Her s-Cr values (eGFR) ​​at discharge were 1.58mg/dL (24 mL/min/1.73m^2^), and those were 1.68 (22), 1.56 (24), 1.47 (26), 1.57 (24), and 1.50 (25) after 1, 3, 6, 12, and 24 months, respectively. Two years after treatment initiation, relapse of vasculitis has not been observed, and immunosuppressive therapy, including glucocorticoid and avacopan, has been discontinued with ongoing observation.

## Discussion

Systemic vasculitis is characterized by dysregulation in adaptive immunity (20, 21); therefore, immunosuppressive therapy remains the main strategy of treatment. Standard therapies include the used of glucocorticoids, cyclophosphamide, rituximab, azathioprine, and avacopan (2, 3). For severe complications, such as DAH, PLEX is often considered to remove pathogenic autoantibodies (ANCA) and inflammatory mediators (22). Indeed, the usefulness of PLEX could be considered in elderly patients with alveolar hemorrhage or severe renal dysfunction (2, 23). These treatments have demonstrated their efficacy and safety in many clinical trials from which older patients were excluded (16, 18).

In older patients, however, immunosuppression increases the risk of infection and may exacerbate comorbid conditions such as diabetes mellitus and hypertension (24). For this group, a better prognosis can be expected by understanding and monitoring the patient’s overall condition and the state of comorbidities during vasculitis treatment. Consequently, dose and duration adjustments are required, but optimal administration varies considerably between individuals, making standardized approaches difficult. In this case, despite her advanced age, our patient had maintained independent living prior to disease onset and did not experience worsening of comorbidities or cognitive decline during vasculitis treatment. Therefore, treatment largely consistent with standard protocols was feasible. Nevertheless, when hypoxemia due to DAH developed, advanced discussions were required regarding limitations of care, including avoidance of invasive mechanical ventilation and confirmation of do-not-attempt-resuscitation (DNAR) preferences. Delirium related to various invasive treatments is frequently encountered in older patients and may compromise adequate therapy. In our case, transient delirium occurred during NPPV and PLEX with hemodialysis therapy, but was successfully managed with minimal sedation, allowing uninterrupted multidisciplinary treatment to continue.

After remission induction, glucocorticoids were tapered according to the reduced-dose regimen described in the PEXIVAS trial and discontinued after 2 years of treatment. The taper milestone was the vasculitis activity, such as BVAS zero (9). We reduced total dose of avacopan by her medical and physical difference. In general, drug metabolism capacity decreases by about 30% in the elderly (25). Furthermore, BMI of the participants of ADVOCATE study was 26, their body surface area would be 1.95 when the average height of the participants would be 175 cm (16). Since the body surface area of our patient was 1.56, we decided to use avacopan at a dose of 20 mg b.i.d. Recently, the efficacy and safety of avacopan in older Japanese patients has recently been reported (14). As standardized protocols for this drug tapering were lacking, avacopan was gradually reduced and discontinued in parallel with glucocorticoids. No relapse has been observed to date and she had regained her ambulatory and independent living during follow-up.

This is a case report of a single elderly patient with severe MPA. The treatment options for an elderly patient, such as prescription dose and duration, and number of PLEX would be case sensitive. Furthermore, it does not propose a standardization for the very elderly. This is a limitation of this study. Nevertheless, when her activities of daily living (ADL) were quantified using Barthel index, her score improved from 50 points upon admission to 60 points upon discharge. Currently, she is able to perform ADL at a level of 80 points.

In conclusion, this case demonstrates that, even in a very elderly patient with MPA complicated by RPGN and DAH, carefully tailored multidisciplinary treatment might induce remission and allow meaningful functional recovery. We provided thorough explanations of her functional reserve, comorbidities, potential adverse effects and patient goals regarding the standard treatment of MPA for a very elderly patient with limited evidence to the patient and her family, and obtained their informed consent before the treatment. She was ultimately able to return to daily life at a level close to that before the onset of MPA.

## Data Availability

The original contributions presented in the study are included in the article/supplementary material. Further inquiries can be directed to the corresponding author.

## References

[1] Nakajima K Kaneko S Usui J Tsuboi N Sugiyama H Maruyama S . Temporal changes of the life and renal prognoses of patients with rapidly progressive glomerulonephritis in Japan, 1989-2019. Clin Exp Nephrol. (2025) 29:937–52. doi: 10.1007/s10157-025-02643-6 40131604 PMC12204914

[2] Hellmich B Sanchez-Alamo B Schirmer JH Berti A Blockmans D Cid MC . EULAR recommendations for the management of ANCA-associated vasculitis: 2022 update. Ann Rheum Dis. (2024) 83:30–47. doi: 10.1136/ard-2022-223764 36927642

[3] Sada KE Nagasaka K Kaname S Nango E Kishibe K Dobashi H . Clinical practice guidelines of the Japan Research Committee of the Ministry of Health, Labour, and Welfare for Intractable Vasculitis for the management of microscopic polyangiitis and granulomatosis with polyangiitis: The 2023 update - secondary publication. Mod Rheumatol. (2024) 34:559–67. doi: 10.1093/mr/road081 37599461

[4] Watts RA Scott DG Jayne DR Ito-Ihara T Muso E Fujimoto S . Renal vasculitis in Japan and the UK--are there differences in epidemiology and clinical phenotype? Nephrol Dial Transplant. (2008) 23:3928–31. doi: 10.1093/ndt/gfn354 18565978

[5] Fujimoto S Watts RA Kobayashi S Suzuki K Jayne DR Scott DG . Comparison of the epidemiology of anti-neutrophil cytoplasmic antibody-associated vasculitis between Japan and the U.K. Rheumatol (Oxford). (2011) 50:1916–20. doi: 10.1093/rheumatology/ker205 21798892

[6] Sada KE Yamamura M Harigai M Fujii T Dobashi H Takasaki Y . Classification and characteristics of Japanese patients with antineutrophil cytoplasmic antibody-associated vasculitis in a nationwide, prospective, inception cohort study. Arthritis Res Ther. (2014) 16:R101. doi: 10.1186/ar4550 24758294 PMC4060546

[7] Jennette JC Falk RJ Bacon PA Basu N Cid MC Ferrario F . 2012 revised international Chapel Hill consensus conference nomenclature of vasculitides. Arthritis Rheum. (2013) 65:1–11. doi: 10.1007/s10157-013-0869-6 23045170

[8] Chevet B Boscato Sopetto G Pagnoux C Specks U Berti A Cornec D . Aging in granulomatosis with polyangiitis and microscopic polyangiitis: from pathophysiology to clinical management. Drugs Aging. (2025) 42:615–31. doi: 10.1007/s40266-025-01210-8 40448791 PMC12254096

[9] Mukhtyar C Lee R Brown D Carruthers D Dasgupta B Dubey S . Modification and validation of the Birmingham vasculitis activity score (version 3). Ann Rheum Dis. (2009) 68:1827–32. doi: 10.1136/ard.2008.101279 19054820

[10] Berden AE Ferrario F Hagen EC Jayne DR Jennette JC Joh K . Histopathologic classification of ANCA-associated glomerulonephritis. J Am Soc Nephrol. (2010) 21:1628–36. doi: 10.1681/asn.2010050477 20616173

[11] Yamagata K Usui J Nagata M Sugiyama H Sada KE Muso E . Histopathological classification of anti-neutrophil cytoplasmic antibody-associated glomerulonephritis in a nationwide Japanese prospective 2-year follow-up cohort study. Clin Exp Nephrol. (2019) 23:387–94. doi: 10.1007/s10157-018-1656-1 30306344

[12] Odler B Riedl R Geetha D Szpirt WM Hawley C Uchida L . The effects of plasma exchange and glucocorticoids on early kidney function among patients with ANCA-associated vasculitis in the PEXIVAS trial. Kidney Int. (2025) 107:558–67. doi: 10.1016/j.kint.2024.11.029 39708998

[13] Walsh M Merkel PA Peh CA Szpirt WM Puechal X Fujimoto S . Plasma exchange and glucocorticoids in severe ANCA-associated vasculitis. N Engl J Med. (2020) 382:622–31. doi: 10.1056/nejmoa1803537 32053298 PMC7325726

[14] Geetha D Pagnoux C Sattui SE Merkel PA Weiner M Draibe J . Efficacy and safety of avacopan in patients aged 65 years and older with ANCA-associated vasculitis: a post hoc analysis of data from the ADVOCATE trial. Rheumatol (Oxford). (2025) 64:3863–71. doi: 10.1093/rheumatology/keaf122 40037556 PMC12107037

[15] Mori K Shirai T Mutoh T Inoue J Fujishima F Kubo S . Drug-induced liver injury related to avacopan therapy. Rheumatol (Oxford). (2025) 64:2533–40. doi: 10.1093/rheumatology/keae689 39672792

[16] Jayne DRW Merkel PA Schall TJ Bekker P Group AS . Avacopan for the treatment of ANCA-associated vasculitis. N Engl J Med. (2021) 384:599–609. doi: 10.1056/nejmoa2023386 33596356

[17] Thietart S Karras A Augusto JF Philipponnet C Carron PL Delbrel X . Evaluation of rituximab for induction and maintenance therapy in patients 75 years and older with antineutrophil cytoplasmic antibody-associated vasculitis. JAMA Netw Open. (2022) 5:e2220925. doi: 10.1001/jamanetworkopen.2022.20925 35802372 PMC9270693

[18] Stone JH Merkel PA Spiera R Seo P Langford CA Hoffman GS . Rituximab versus cyclophosphamide for ANCA-associated vasculitis. N Engl J Med. (2010) 363:221–32. doi: 10.1056/nejmoa0909905 20647199 PMC3137658

[19] Turner-Stokes T Sandhu E Pepper RJ Stolagiewicz NE Ashley C Dinneen D . Induction treatment of ANCA-associated vasculitis with a single dose of rituximab. Rheumatol (Oxford). (2014) 53:1395–403. doi: 10.1093/rheumatology/ket489 24609057

[20] Nakazawa D Masuda S Tomaru U Ishizu A . Pathogenesis and therapeutic interventions for ANCA-associated vasculitis. Nat Rev Rheumatol. (2019) 15:91–101. doi: 10.1038/s41584-018-0145-y 30542206

[21] Kronbichler A Bajema IM Bruchfeld A Mastroianni Kirsztajn G Stone JH . Diagnosis and management of ANCA-associated vasculitis. Lancet. (2024) 403:683–98. doi: 10.1016/s0140-6736(23)01736-1 38368016

[22] Fussner LA Flores-Suarez LF Cartin-Ceba R Specks U Cox PG Jayne DRW . Alveolar hemorrhage in antineutrophil cytoplasmic antibody-associated vasculitis: results of an international randomized controlled trial (PEXIVAS). Am J Respir Crit Care Med. (2024) 209:1141–51. doi: 10.1164/rccm.202308-1426oc 38346237 PMC11092964

[23] Kidney Disease: Improving Global Outcomes AVWG . KDIGO 2024 clinical practice guideline for the management of antineutrophil cytoplasmic antibody (ANCA)-associated vasculitis. Kidney Int. (2024) 105:S71–S116. doi: 10.1016/j.kint.2023.10.008 38388102

[24] Sada KE Ohashi K Asano Y Hayashi K Morishita M Watanabe H . Treatment-related damage in elderly-onset ANCA-associated vasculitis: safety outcome analysis of two nationwide prospective cohort studies. Arthritis Res Ther. (2020) 22:236. doi: 10.1186/s13075-020-02341-6 33046139 PMC7552473

[25] Klotz U . Pharmacokinetics and drug metabolism in the elderly. Drug Metab Rev. (2009) 41:67–76. doi: 10.1080/03602530902722679 19514965

