# Correction: Successful reintegration of a very elderly patient with rapidly progressive glomerulonephritis due to microscopic polyangiitis after multidisciplinary treatment: a case report

**DOI:** 10.3389/fneph.2026.1895403

**Published:** 2026-06-19

**Authors:** Tatsuo Tsukamoto, Yui Fukuda, Kaoru Ohue, Atsuto Niwa, Sanae Ogura, Takuya Ishimura, Takaya Handa, Tomomi Endo, Takeshi Matsubara

**Affiliations:** 1 Department of Nephrology and Dialysis, Medical Research Institute Kitano Hospital, PIIF Tazuke-Kofukai, Osaka, Japan; 2 Center for Preventive Medicine, Medical Research Institute Kitano Hospital, PIIF Tazuke-Kofukai, Osaka, Japan

**Keywords:** elderly population, microscopic polyangiitis, multidisciplinary approach, rapidly progressive glomerulonephritis, reintegration

There was a mistake in **Table 1** as published. The table heading and example text are displayed incorrectly. The corrected **Table 1** appears below.

**Table 1:** Laboratory data on admission.

Urinalysis			Blood chemistry		
Protein	2+		AST	30	U/L
Occult blood	3+		ALT	10	U/L
Urobilinogen	±		LDH	169	U/L
Gradient	1.015		ALP	87	U/L
RBC	>100	/HPF	g-GTP	41	U/L
WBC	50-99	/HPF	T-Bil	0.6	mg/dL
NAG	60.5	IU/L	CK	17	U/L
uPCR	1425.6	mg/gCr	Glucose	105	mg/dL
CBC			Total Protein	5.7	g/dL
RBC	341	x10^4^/mL	Albumin	1.8	g/dL
Hb	9.5	g/dL	T-Chol	132	mg/dL
Ht	29.2	%	LDL-Chol	58	mg/dL
WBC	12,800	/mL	BUN	27.6	mg/dL
PLT	28.4	x10^4^/mL	UA	8.1	mg/dL
Immunology			Cr	2.18	mg/dL
IgG	1297	mg/dL	Na	133	mEq/L
IgA	484	mg/dL	K	3.7	mEq/L
IgM	62	mg/dL	Cl	97	mEq/L
C3	97	mg/dL	Ca	7.8	mg/dL
C4	17	mg/dL	P	3.5	mg/dL
MPO-ANCA	115	U/mL	CRP	19.49	mg/dL
PR3-ANCA	<1.0	U/mL			
anti-GBM Ab	<2.0	U/mL			
ANA	x40				

PCR, protein creatinine ratio.

The original version of this article has been updated.

